# Recent Advances of Persistent Luminescence Nanoparticles in Bioapplications

**DOI:** 10.1007/s40820-020-0404-8

**Published:** 2020-03-10

**Authors:** Shuqi Wu, Yang Li, Weihang Ding, Letong Xu, Yuan Ma, Lianbing Zhang

**Affiliations:** 1 School of Life Sciences, Key Laboratory of Space Bioscience and Biotechnology, Northwestern Polytechnical University, Xi’an, 710072 People’s Republic of China; 2 School of Materials Science and Engineering, Northwestern Polytechnical University, Xi’an, 710072 People’s Republic of China

**Keywords:** Persistent luminescence nanoparticles, Biosensing, Bioimaging, Cell tracking, Cancer therapy

## Abstract

Persistent luminescence phosphors are a novel group of promising luminescent materials with afterglow properties after the stoppage of excitation. In the past decade, persistent luminescence nanoparticles (PLNPs) with intriguing optical properties have attracted a wide range of attention in various areas. Especially in recent years, the development and applications in biomedical fields have been widely explored. Owing to the efficient elimination of the autofluorescence interferences from biotissues and the ultra-long near-infrared afterglow emission, many researches have focused on the manipulation of PLNPs in biosensing, cell tracking, bioimaging and cancer therapy. These achievements stimulated the growing interest in designing new types of PLNPs with desired superior characteristics and multiple functions. In this review, we summarize the works on synthesis methods, bioapplications, biomembrane modification and biosafety of PLNPs and highlight the recent advances in biosensing, imaging and imaging-guided therapy. We further discuss the new types of PLNPs as a newly emerged class of functional biomaterials for multiple applications. Finally, the remaining problems and challenges are discussed with suggestions and prospects for potential future directions in the biomedical applications.

## Highlights

- Comprehensive summary on properties, persistent luminescence mechanism and synthesis of persistent luminescence nanoparticles.
- Unique properties and advantages of persistent luminescence nanoparticles for chem/biosensing, bioimaging and imaging-guided therapy.
- New organic and polymeric persistent luminescence nanoparticles with long afterglow lifetime for in vivo optical imaging.

## Graphical abstract

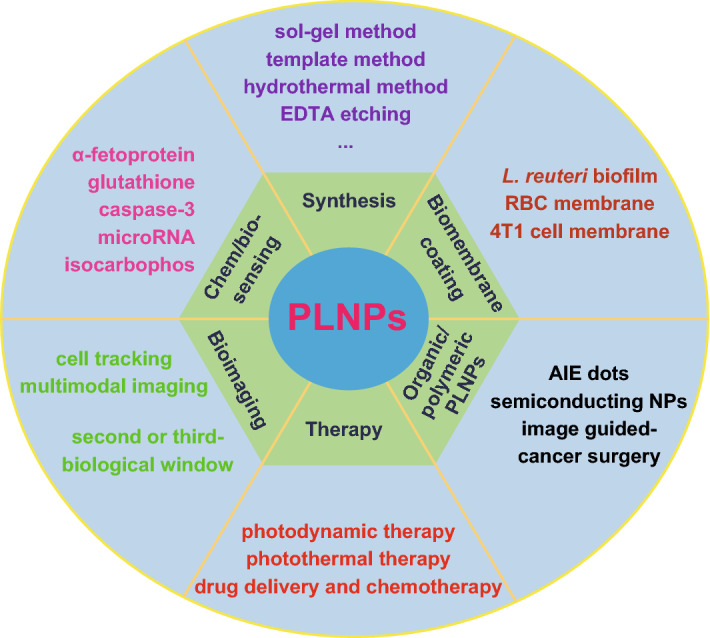

## Introduction

Afterglow or persistent luminescence materials can store energy from UV light, visible light, X-ray or some other excitation sources and then gradually release it by a photonic emission [1–3]. The persistent emission can last for minutes, hours or even days after the stoppage of the excitation. The discovery of persistent luminescence phenomenon dates back to the Song Dynasty of China. Some paintings or so-called luminous pearls had incomprehensible magic to glow in the dark [4]. At the beginning of the seventeenth century, an Italian shoemaker first described the famous Bologna stone which emitted yellow to orange afterglow in darkness. Later, the natural impurities of BaS were found to play an important role in this persistent luminescence phenomenon. In 1996, Matsuzawa et al. reported a new phosphor of metallic oxide (SrAl_2_O_4_:Eu^2+^, Dy^3+^) which showed extremely bright and long phosphorescence [5]. Since then, persistent luminescence materials have been rapidly developed and lots of phosphors with different matrixes and doped ions have been reported. Up to now, inorganic metal compounds, metal–organic frameworks, some organic composites and polymers have been found to have long afterglow properties.

The unique properties of persistent luminescence materials mainly come from two kinds of active centers involved: the emitter centers and the trap centers [6–8]. The emitter centers can emit radiation after excitation. So, the emission wavelength of a persistent luminescence phosphor depends upon the emitter. The trap centers are formed due to impurities, lattice defects, or various co-dopants. They usually do not emit radiation, but store the excitation energy for some time and then gradually release it to the emitters by thermal or other physical activation. Therefore, the persistent intensity and time are mainly determined by the traps [2]. In design of persistent luminescence materials, a suitable emitter center and a proper host that can create appropriate traps and release long-lasting persistent luminescence (PL) should be considered [6].

There are persistent luminescence materials for each of the primary colors. Theoretically, we can synthesize persistent luminescence materials emitting any color by adjusting the chemical components. The most widely used and studied matrixes include silicates [9], gallium oxides [10], gallogermanates [11], aluminates [12] and so on [13–16]. Among them, CaAl_2_O_4_:Eu^2+^, Nd^3+^ and SrAl_2_O_4_:Eu^2+^, Dy^3+^ with overwhelming strong and long-lasting PL have been commercialized and widely used in various fields [2, 17]. With the fast development of persistent luminescence materials in recent years, some new phosphors with special compositions have been developed for multiple bioapplications, such as Ca_3_Ga_2_Ge_3_O_12_:Cr^3+^, Li_5_Zn_8_Al_5_Ge_9_O_36_:Cr^3+^ and (Li, Na)_8_Al_6_Si_6_O_24_(Cl, S)_2_:Ti^3+^ [18–20]. Tu et al. reported the rare-earth ions (Pr^3+^, Nd^3+^ and Gd^3+^)-doped Li_2_ZnGeO_4_ with better afterglow properties due to their larger defect density values [21]. Furthermore, up-converting ions Yb^3+^-Er^3+^-incorporated Zn_3_Ga_2_SnO_8_:Cr^3+^ showed an obvious near-infrared (NIR)-emitting PL after the stoppage of 980-nm laser irradiation [22]. These upconversion-persistent luminescence materials combined the advantages of both upconversion and persistent luminescence, paving a new way for biomedical applications [23–25].

Persistent luminescence materials emitting visible light have been successfully commercialized and widely used in security signs, traffic signs, dials, luminous paints and so on. In recent years, the deep-trap persistent luminescence materials with the unique characters of energy storage and controllable photon release showed great promising potential in the application of information storage, multilevel anticounterfeiting and advanced displays [26–28]. Although the intensity and afterglow time of nanosized persistent luminescence nanoparticles (PLNPs) were much lower than the bulk materials [29, 30], PLNPs have been widely investigated as optical probes in bioimaging and biosensing due to the nanoeffects, the efficient cell penetration ability, the better biocompatibility, etc. [31]. Unlike conventional fluorescent probes (e.g., organic dyes, quantum dots, or upconversion nanoparticles) with very short lifetime, PLNPs can be used without constant in situ excitation. The persistent luminescence signals can be easily captured in the bioluminescence mode on imaging instruments. Furthermore, Cr^3+^-doped PLNP with NIR emission open an extensive application in vivo, as the emission matches well with the first biological window (650–950 nm) and they can be re-activated with white or red LED light [3, 32–34]. The absence of autofluorescence background interferences and the NIR emission gave a high signal-to-noise ratio (SNR) and a better in vivo penetration depth. Through size and emission regulation, as well as surface modifications, PLNPs have been extensively used in biosensing, bioimaging and imaging-guided therapy as a new generation of advanced optical materials [35].

**Scheme 1:**
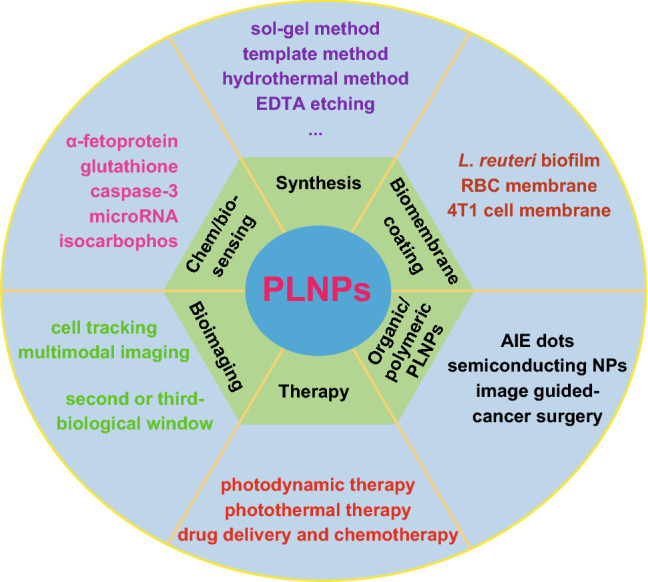
Summary of advanced PLNPs for bioapplications

Besides the most studied biosensing and bioimaging, scientists developed many novel PLNPs-based nanocomposites for special applications, such as antibacterium, latent fingerprint imaging and photocatalytic pollutant degradation [36–38]. In recent years, Nd^3+^-doped PLNPs with the NIR emission located in the second (1000–1400 nm) or the third (1500–1800 nm) biological window have been developed to achieve high imaging depth and sensitivity [39, 40]. What is more, some organic PLNPs show promising advantages for in vivo afterglow imaging and detection [41, 42]. All these achievements make PLNPs novel multifunctional tools in bioapplications [43, 44]. A number of excellent reviews have already been published with the focus on the synthesis, surface engineering and biological applications of PLNPs. In 2017, Wang et al. summarized the recent achievements in biosensing, bioimaging and cancer therapy of PLNPs [45]. Sun et al. summarized their systematic achievements in the bioapplications of PLNPs from biosensing/bioimaging to theranostics. They developed target-induced formation or interruption of fluorescence resonance energy transfer (FRET) systems for biosensing and imaging of cancer biomarkers without autofluorescence interferences. They decorated targeting ligands or specific functional groups on PLNPs for tumor-targeted imaging, multimodal imaging and cancer therapy. They also proposed the design principle and comprehensive strategies for guiding and promoting further development of PLNPs in biological science and medicine [46]. Liang et al. summarized the design and applications of NIR-emitted PLNPs and emphasized their luminescence mechanism [47]. However, a review summarizing recent advances in synthesis methods, new types of organic/polymeric composition, biomembrane coating techniques, biosafety and bioapplications of PLNPs is lacking. The aim of this review is to present a comprehensive discussion on the synthesis and functionalization of PLNPs and the recent progress on PLNPs-based biosensing, bioimaging and therapy applications. This review further explores the future developments of PLNPs on the clinical applications (Scheme 1).

## Typical Synthetic Procedures for PLNPs

In the past decade, various kinds of PLNPs have been synthesized by the solid-state synthetic methods with less control over the size and the shape of the products. As a result, the endured long reaction time and high annealing temperature of the synthesis make the phosphors bulky and irregular, which limit the usage in biomedical fields [34]. Therefore, new size- and shape-controllable synthetic methods have been developed to prepare nanosized PLNPs, such as sol–gel methods, template methods, hydrothermal/solvothermal methods and other wet-chemical synthesis methods [48–57]. In this section, we will discuss these typical synthetic approaches for PLNPs.

### Sol–Gel Methods

Compared with the solid-state synthetic methods, the sol–gel methods can offer better purity, homogeneity and yield stoichiometric powders at relatively lower annealing temperature [58]. The synthetic conditions, including the reaction time, pH, temperature, concentration of the surfactants, chemical composition, etc., can be flexibly adjusted with the wet chemical synthesis. Abdukayum et al. reported the synthesis of NIR-emitting PLNPs by a citrate sol–gel method without the need for a reducing atmosphere. The authors assessed the effects of pH, annealing temperature, sinter time and composition on the luminescence intensity of PLNPs. It was found that the optimal pH of the starting solution is about 5. The increase in calcination time can promote the NIR persistent luminescence intensity at 1000 °C with the proper composition of Zn_2.94_Ga_1.96_Ge_2_O_10_:Cr_0.01_Pr_0.03_ [33]. The resulting powder is usually in micrometer size scale, highly agglomerated and morphologically irregular. The main reason is due to the uncontrol over the nanoparticle growth stage and the agglomeration during the calcination with high temperature [59, 60]. Now, the wet grinding and selective sedimentation method have been used to isolate the smallest nanoparticles from the bulk materials, which suffers greatly from the trivial and time-consuming procedures with very low yield. The synthesized nanoparticles are usually non-spherical with a polydisperse size distribution in the range of 40–150 nm,which undoubtedly limits their advanced applications in biomedical fields.

### Template Methods

Zhang and co-workers reported several works about the synthesis of PLNPs by a template method using mesoporous silica nanospheres (MSNs) [61–63]. MSNs can serve as both the morphology-controlling templates and the silicon source of some silicate PLNPs. The metal ions were impregnated in the mesopores of MSNs templates, followed by the annealing under certain conditions to form PLNPs with uniform morphologies and narrow size distributions. The template method can be easily transferred to synthesize PLNPs with different composition. The diameter and morphologies can be flexibly controlled by changing the MSNs templates [64]. However, up to now, MSNs was the only template that has been successfully used for synthesis of PLNPs. In addition, the high calcination temperature could destroy the surface functional groups, which may lead to the undesirable accumulation and poor dispersibility of PLNPs. As a result, the novelty and biomedical application of synthesizing functional PLNPs by template method are restricted.

### Synthesis of Monodispersed PLNPs

The controlled synthesis of monodispersed and small sized PLNPs is essential for extended bioimaging and therapeutic applications, as large hydrodynamic-sized (> 100 nm) PLNPs are often quickly taken up and trapped in the reticuloendothelial system (RES). Therefore, it remains challenging to create nanosized PLNPs with high biocompatibility. The hydrothermal/solvothermal method, the non-aqueous sol–gel methods, the bi-phasic synthesis methods and other synthetic procedures are used to prepare monodispersed PLNPs [65–67].

**Fig. 1:**
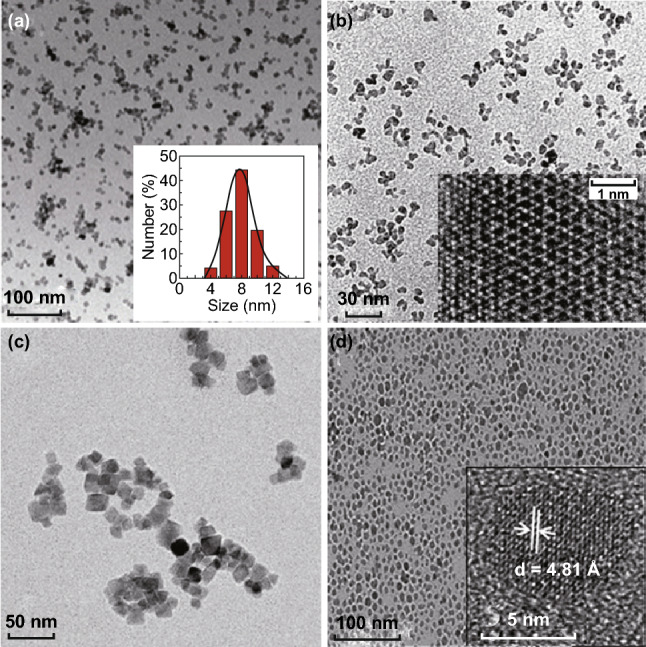
The morphology of the monodispersed and small-sized PLNPs. **a** Direct aqueous-phase synthesis of sub-10-nm PLNPs. Reproduced with permission from Ref. [54]. Copyright 2015 American Chemical Society. **b** Synthesis of ultra-small PLNPs using a non-aqueous sol–gel method. Reproduced with permission from Ref. [50]. Copyright 2015 Wiley–VCH Verlag GmbH & Co. KGaA, Weinheim. **c** Silica shell-assisted synthetic route for synthesizing monodispersed PLNPs. Reproduced with permission from Ref. [67]. Copyright 2016 Tsinghua University Press and Springer-Verlag Berlin Heidelberg. **d** Synthesis of sub-10-nm PLNPs using a bi-phasic route. Reproduced with permission from Ref. [65]. Copyright 2012 The Royal Society of Chemistry

In 2015, Li and co-workers first developed a direct aqueous-phase chemical synthesis route of NIR PLNPs (Fig. 1a). Their method leads to monodispersed PLNPs with the diameter as small as ca. 8 nm which present enhanced renewable NIR persistent luminescence in vivo. More importantly, such sub-10-nm PLNPs are readily functionalized and can be stably dispersed in aqueous solutions and cell culture medium for biological applications. Such nanocrystals possess superior red light renewable persistent luminescence both in vitro and in vivo, which can broad their use in photonics and biophotonics as advanced miniature “luminous pearls” [54]. Teston and co-workers designed a facile one-pot synthesis of ultra-small (6 nm) PLNPs by using a non-aqueous sol–gel method assisted with microwave irradiation (Fig. 1b). This strategy allows the control over the crystal growth by using the microwave heating as the energy source. The synthesized PLNPs were then easily surface-modified with polyethylene glycol phosphonate moieties [50]. Recently, Zou et al. have established a robust and controllable three-step strategy involving the coating/etching of the SiO_2_ shell to synthesize monodispersed PLNPs (~ 15 nm) without any agglomeration (Fig. 1c). This advanced strategy provides an ideal route to fabricate novel optical materials with excellent size distribution, dispersity and biocompatibility [67]. Srivastava et al. synthesized a sub-10-nm Cr-doped ZnGa_2_O_4_ nanoparticles by a bi-phasic synthesis route through the hydrolysis of inorganic salts in a water–toluene system (Fig. 1d). This synthesis strategy can control the particle size and shape by the slow nucleation process [65]. All these aforementioned methods provide feasible ways to synthesize monodispersed PLNPs and can be extended to synthesize other functional metal oxide nanoparticles.

**Fig. 2:**
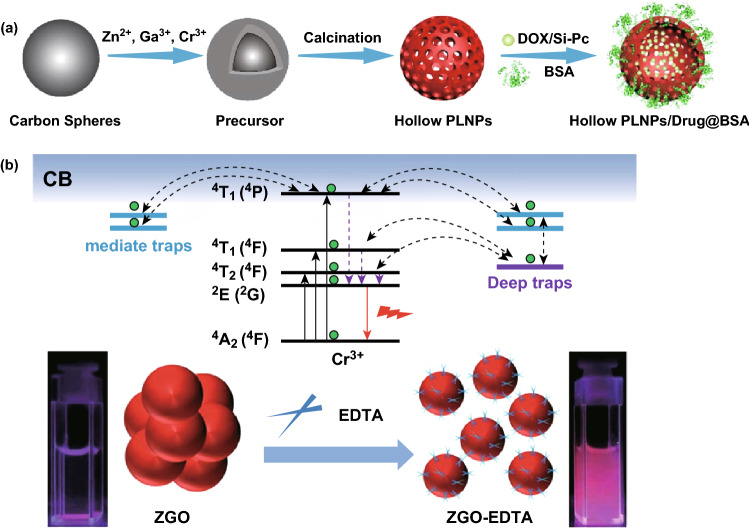
**a** Synthesis and functionalization of the hollow NIR PLNPs. Reproduced with permission from Ref. [70]. Copyright 2018 American Chemical Society. **b** Regulating the traps, size and aqueous dispersibility of PLNPs by EDTA etching. Reproduced with permission from Ref. [72]. Copyright 2018 The Royal Society of Chemistry

In a similar fashion, Li and co-workers report the synthesis of 5-nm PLNPs with NIR emission at 800 nm via a direct aqueous-phase synthesis method [66]. This one-step hydrothermal synthetic route can easily produce PLNPs with abundant surface hydroxyl groups, avoiding complicated surface modification steps. Other synthesis routes using pulsed laser ablation, vacuum-annealing and surfactant-aided hydrothermal steps, etc., can provide ultra-bright monodispersed PLNPs with the super-long NIR persistent luminescence for in vivo bioapplications [68, 69]. For example, Wang et al. reported novel size-tunable hollow-structured PLNPs by crystallizing the immobilized parent ions on the carbon spheres and calcining. The large hollow cavity of PLNPs allows the high loading of chemical drugs and photosensitizers which can be used for chemo/photodynamic therapies (Fig. 2a) [70].

It is generally accepted that small size usually leads to short persistent luminescence and low quantum yield. To synthesize monodispersed small-sized PLNPs with bright and super-long afterglow remains challenging. Wang et al. reported the zinc gallogermanate PLNPs (Zn_1+*x*_Ga_2−2*x*_Ge_*x*_O_4_:Cr, 0 ≤ *x* ≤ 0.5) with the composition-dependent size distribution and persistent luminescence. The intensity and decay time of the persistent luminescence can be fine-tuned by simply changing the formula [71]. Moreover, Wang and co-workers demonstrated a simultaneous control of the traps, size and aqueous dispersibility via a simple ethylenediaminetetraacetate (EDTA) etching. The resulting PLNPs-EDTA showed the suitable mediate/deep traps, a fine aqueous dispersibility and the super-long bright afterglows (Fig. 2b) [72]. The reported methods produced strong NIR-emitting and broadened the use of PLNPs in various research fields. Nonetheless, there are still many works needed to be done to deal with the tradeoff between the size and the persistent luminescence performance.

### Biomembrane Bioinspired PLNPs

**Fig. 3:**
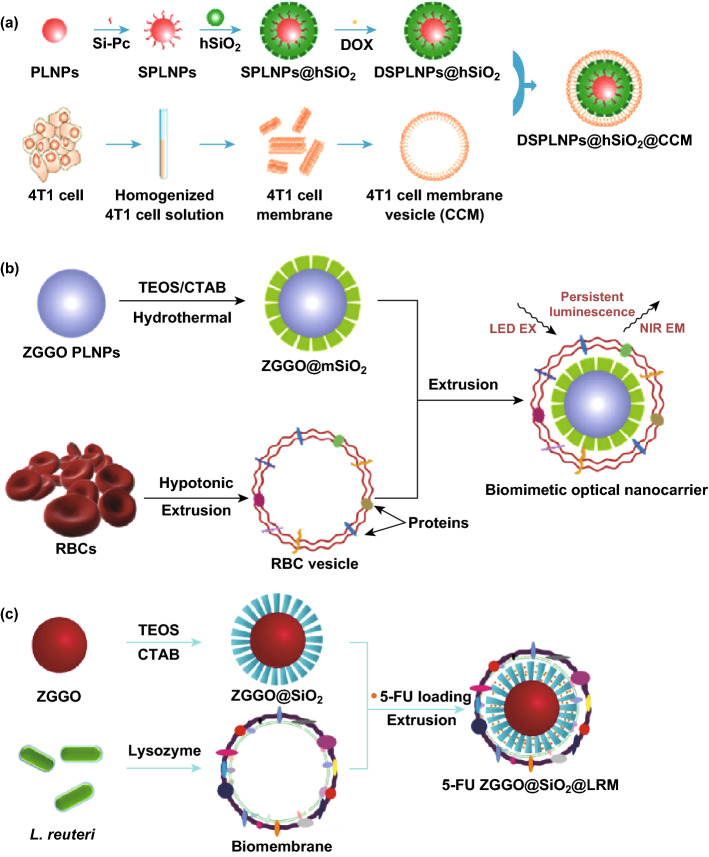
**a** PLNPs nanocomposite was coated with 4T1 tumor cell membrane for PL imaging-guided chemo/photodynamic therapy of metastasis. Reproduced with permission from Ref. [73]. Copyright 2018 American Chemical Society. **b** PLNPs nanocomposite was coated with red blood cell membrane for in vivo long-circulating bioimaging and drug delivery. Reproduced with permission from Ref. [74]. Copyright 2018 Elsevier Ltd. **c** PLNPs nanocomposite was coated with *Lactobacillus reuteri* biofilm for colorectal cancer imaging and orally administrated chemotherapy. Reproduced with permission from Ref. [75]. Copyright 2019 American Chemical Society

In order to avoid the recognition and phagocytosis by the immune system, multifunctional surface modification of nanodrug delivery systems was developed, including hydrophilic polymer modification, liposome encapsulation, tumor microenvironment responding strategies and so on. Li et al. reported the 4T1 tumor cell membrane-coated PLNPs-based nanocomposite for effective metastasis theranostic (Fig. 3a) [73]. This cancer cell membrane coating provided the nanocomposite with metastasis targeting ability and prevents the drug leakage. Due to the intrinsic biocompatibility and non-immunogenicity, red blood cell (RBC) membrane has also been successfully utilized to coat PLNPs-based nanocomposite as the biomimetic modifier (Fig. 3b) [74]. The RBC membrane-coated biomimetic nanocarriers showed a super-long persistent luminescence with the red-light renewability, a monodispersed nanosize and an excellent biocompatibility, which are suitable for in vivo long-circulating bioimaging and concomitant drug delivery. Moreover, *Lactobacillus reuteri* biofilm (LRM) was coated on the PLNPs nanocomposite for orally administered PL bioimaging and delivering colorectal cancer chemotherapeutic drug (Fig. 3c). This novel drug delivery system could protect the drugs from the gastric acid digestion and localize colorectum, which may give new prospects for oral drugs delivery [75]. These biomembrane-modified PLNPs nanoplatforms offer promising potential for targeted cancer imaging and therapy.

## PLNPs for Chemo/Biosensing

High target selectivity and sensitivity of chem/biosensing probes play an important role in chem/biomedical detections. For example, the detection of tumor biomarkers, metabolites, biomolecules and other fundamental signal parameters in living cells is essential for disease theranostic and systematic studies on cell activities. Because of the elimination of the in situ excitation, PLNPs with long-lasting afterglow nature allow the chem/biosensing without background noise interferences. In particular, the NIR-emitting PLNPs showed the high penetration depth in biological tissues, the good photo- and chemical stability and the low toxicity which make them exceptionally favorable in chem/biosensing processes. PLNPs can be easily modified with various functional groups for the target detection and numerous efforts were devoted for chem/biosensing based on these functional PLNPs (Table 1) [76]. Among these works, the FRET pathways play a crucial role in the detection process. The following paragraphs will focus on several detection applications utilizing PLNPs.

**Table 1:** Chem/biosensing based on functional PLNPs

Composition of PLNPs	Surface functionalization	Conjugation	Target	Detection mode	Limit of detection	Refs.
Ca_1.86_Mg_0.14_ZnSi_2_O_7_:Dy^3+^, Eu^2+^	PEI	Ab-AuNPs	α-Fetoprotein (AFP)	Off–on	0.41 μg L^−1^	[77]
Sr_2_MgSi_2_O_7_:Eu^3+^, Dy^3+^	Hydroxylated	CoOOH nanoflakes	Ascorbic acid	Off–on	2.20 μM	[78]
Sr_2_MgSi_2_O_7_:Eu^3+^, Dy^3+^	Hydroxylated	MnO_2_ nanosheets	Glutathione	Off–on	0.83 μM	[79]
Sr_1.6_Mg_0.3_Zn_1.1_Si_2_O_7_:Eu^2+^, Dy^3+^	FITC-labeled substrate peptide	–	Caspase-3	On–off	2.4 × 10^5^ unit mL^−1^	[80]
Sr_1.6_Mg_0.3_Zn_1.1_Si_2_O_7_:Eu^2+^, Dy^3+^	FITC-labeled DNA	–	MicroRNA-21	Off–on	0.26 pM	[80]
Sr_1.6_Mg_0.3_Zn_1.1_Si_2_O_7_:Eu^2+^, Dy^3+^	FITC-labeled aptamer	–	Platelet-derived growth factor (PDGF)	On–on	2.57 pM	[80]
Ca_1.86_Mg_0.14_ZnSi_2_O_7_:Dy^3+^, Eu^2+^	PSA antibody	Rhodamine B-bonded PSA antibody	Prostate specific antigen (PSA)	On–on	0.09 μg L^−1^	[81]
SrMgSi_2_O_6_:Eu^3+^, Dy^3+^	–	–	Dopamine	On–off	0.78 μM	[83]
Zn_2_GeO_4_:Mn^2+^	Lysozyme-binding aptamer	Black-hole-quencher-labeled DNA (BHQ-DNA)	Lysozyme	Off–on	4.6 nM	[166]
Zn_2_GeO_4_:Mn	–	Gold nanoparticles–aptamer complex	Isocarbophos	On–off	7.1 μg L^−1^	[167]
${\text{Cr}}_{0.004}^{3 +}{:}{\text{ZnGa}}_{2} {\text{O}}_{4}$	Au nanoparticles	Cy5.5-KGPNQC-SH	Fibroblast activation protein-alpha (FAPα)	Off–on	115 pM	[168]
Zn_1.25_Ga_1.5_Ge_0.25_O_4_:0.5%Cr^3+^	LTA antibody	–	Foodborne probiotics	On–on	–	[84]
ZnGa_2_O_4_:Cr^3+^	Insulin-binding aptamer	Au complex	Insulin	Off–on	2.06 pM	[169]
ZnGa_2_O_4_:Cr^3+^	–	–	Hemoglobin	On–off	0.13 nM	[170]
Sr_2_Al_14_O_25_:Eu^2+^, Dy^3+^	–	–	antibiotics	On–off	5 nM	[171]
Sr_2_Al_14_O_25_:Eu^2+^, Dy^3+^	–	–	2,4,6-Trinitrophenol	On–off	10 nM	[171]
Zn_2_GeO_4_:Mn	Single-stranded DNAs	Black-hole-quencher-labeled DNAs	Bladder cancer-related miRNA	Off–on	26.3 fM	[82]
ZnGa_2_O_4_:Cr^3+^	Polyethyleneimine	Dithiothreitol-coated gold nanorods	Arsenic(III)	Off–on	55 nM	[172]

**Fig. 4:**
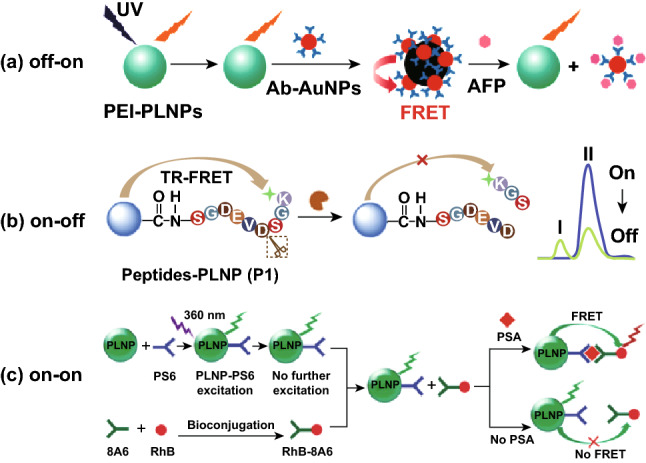
Schematic illustration of PLNPs-based biosensing. **a** FRET inhibition assay for AFP based on the PL quenching of PEI-PLNPs by antibody (Ab)-AuNPs. Reproduced with permission from Ref. [77]. Copyright 2010 American Chemical Society. **b** Detection strategies of caspase-3 protease by using caspase-specific peptide-functionalized PLNPs probe. Reproduced with permission from Ref. [80]. Copyright 2015 Elsevier Ltd. **c** Design for PSA detection using PLNPs-based FRET immunoassay. Reproduced with permission from Ref. [81]. Copyright 2015 The Royal Society of Chemistry

The sensitive and selective sensing of biomarkers in biological environments is crucial for an efficient clinical diagnosis. Compared with the conventional fluorescent sensors based on dyes or QDs, PLNPs can provide noninvasive sensing both in vitro and in vivo and improve the detection limit by the elimination of the autofluorescence interferences. In 2011, Wu and co-workers established a FRET inhibition assay for α-fetoprotein (AFP) using water-soluble functionalized PLNPs (Ca_1.86_Mg_0.14_ZnSi_2_O_7_:Dy^3+^, Eu^2+^). The PLNPs were coated with polyethyleneimine and then conjugated with AFP antibody-coated gold nanoparticles. Au nanoparticles were served as the quencher due to their high molar adsorption coefficient. This highly sensitive and specific persistent photoluminescence probe can detect AFP in serum samples and real-time image the excreted AFP during the cancer cell growth (Fig. 4a) [77].

Similarly, Li and co-workers reported two works about PLNPs-based detection of GSH and ascorbic acid via FRET. PLNPs were used as the luminescence unit and the CoOOH nanoflakes or the MnO_2_ nanoparticles as the quencher. The luminescence of PLNPs can be restored in the presence of ascorbic acid, as the CoOOH quencher was reduced to Co^2+^ [78]. For GSH detection, the presence of GSH reduced MnO_2_ to Mn^2+^ which restored the luminescence [79]. These approaches provide effective platforms for detecting and imaging reactive species in living cells and tissues.

The aforementioned strategies use the nanoparticles as the persistent luminescence quenchers for “off–on” FRET detection. In addition, fluorescent dyes can also serve as luminescence quenchers or emit at different wavelength. Ju’s group established three kinds of PLNPs-based nanoplatforms assembled by covalently binding fluorescent dye (FITC)-labeled peptides or DNA to carboxyl-modified PLNPs for the efficient detection of caspase-3 (Fig. 4b), microRNA and platelet-derived growth factor (PDGF) protein [80]. Wu et al. established a novel FRET immunoassay based on PLNPs for the prostate specific antigen (PSA) detection. The PLNPs conjugated with mouse monoclonal PSA antibody were employed as the energy donor, while Rhodamine B (RB)-bonded another PSA antibody was chosen as the energy accepter. PSA-mediated FRET from the modified PLNPs to RB resulted in the increase in the intensity ratio of RB (at 585 nm) to PLNPs (at 524 nm) with the increase in PSA concentration, which allow the efficient detection of PSA in serum and cell extracts (Fig. 4c) [81].

**Fig. 5:**
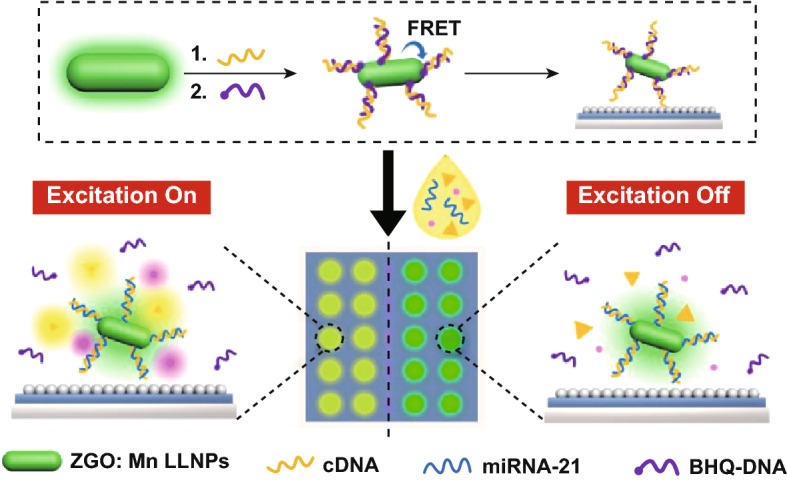
Time-gated detection of bladder cancer-related miRNA-21 by the developed PLNPs-based biochip. Reproduced with permission from Ref. [82]. Copyright 2019 American Chemical Society

The impurities and other analytes in the complex samples may cause the high background noise and hamper the detection sensitivity and accuracy. For example, urine samples contain many kinds of small molecules, proteins and nucleic acids. Wang et al. published a PLNPs-based biochip with the enhanced persistent luminescence signals and the ultra-low autofluorescence background interferences for bladder cancer-related miRNA-21 detection in urine (Fig. 5). Using the time-gated luminescence of PLNPs, the detection sensitivity was significantly improved and a detection limit of 26.3 fM was achieved [82]. This sensitive PLNPs-based detection of the disease-related biomarkers in patient urines can open up a new avenue in painless and noninvasive diagnosis.

The PLNPs-based nanoplatform was employed to detect other important parameters and biomolecules, such as dopamine, temperature, avidin and foodborne probiotics [83–86]. Furthermore, PLNPs-based detection can be produced as the assay reporters in the lateral flow assays [87]. The sensitive and selective detection without background interferences in complex biological samples is urgently needed for medical diagnosis, therapy monitoring and health management. For instance, the detection of heavy metal ions (Hg^2+^, Pb^2+^, etc.), reactive oxygen species (ROS) and high toxic molecules plays an important role in life sciences. Although biosensing based on PLNPs has been reported in many publications, more efforts need to be done before the clinical use.

## PLNPs for Bioimaging

PLNPs were first used by Scherman and co-workers for in vivo imaging [88]. PLNPs without the continuous in situ excitation can efficiently eliminate the background autofluorescence from animal tissues in bioimaging, leading to the significant improvement of the imaging sensitivity and the signal-to-noise ratio (SNR). With the large surface-to-volume ratio, surface modifications can be easily achieved by silica coating, polymer linking, biomolecules conjugation and so forth, after which PLNPs are ready to be employed into extensive bioimaging applications. Compared to conventional in vivo optical imaging probes, PLNPs possess high photostability, superior SNR and excellent biocompatibility and can be directly used in the commercially available imaging systems. Importantly, the long afterglow and red-light renewable capability permit PLNPs to be used for long-term in vivo bioimaging applications [44, 89, 90].

### Cellular Imaging and Tracking

PL imaging allows noninvasive tracking and imaging for visualizing various biological processes of migrating cells, which might be crucial for the biological fate and progression of some diseases. Up to now, imaging of Raw 264.7 macrophages (a phagocytic cell line), stem cells, breast cancer cells, J774A.1 macrophages have been widely studied by labeling with PLNPs [91–95]. Maldiney et al. and Chuang et al. reported the noninvasive long-term in vivo tracking of RAW 264.7 cells with PLNPs. The RAW cells can be efficiently labeled with PLNPs by simple incubation. As the NIR-emitting light has the better penetration, PLNPs labeling can track RAW cells during cell homing process in vivo [3].

**Fig. 6:**
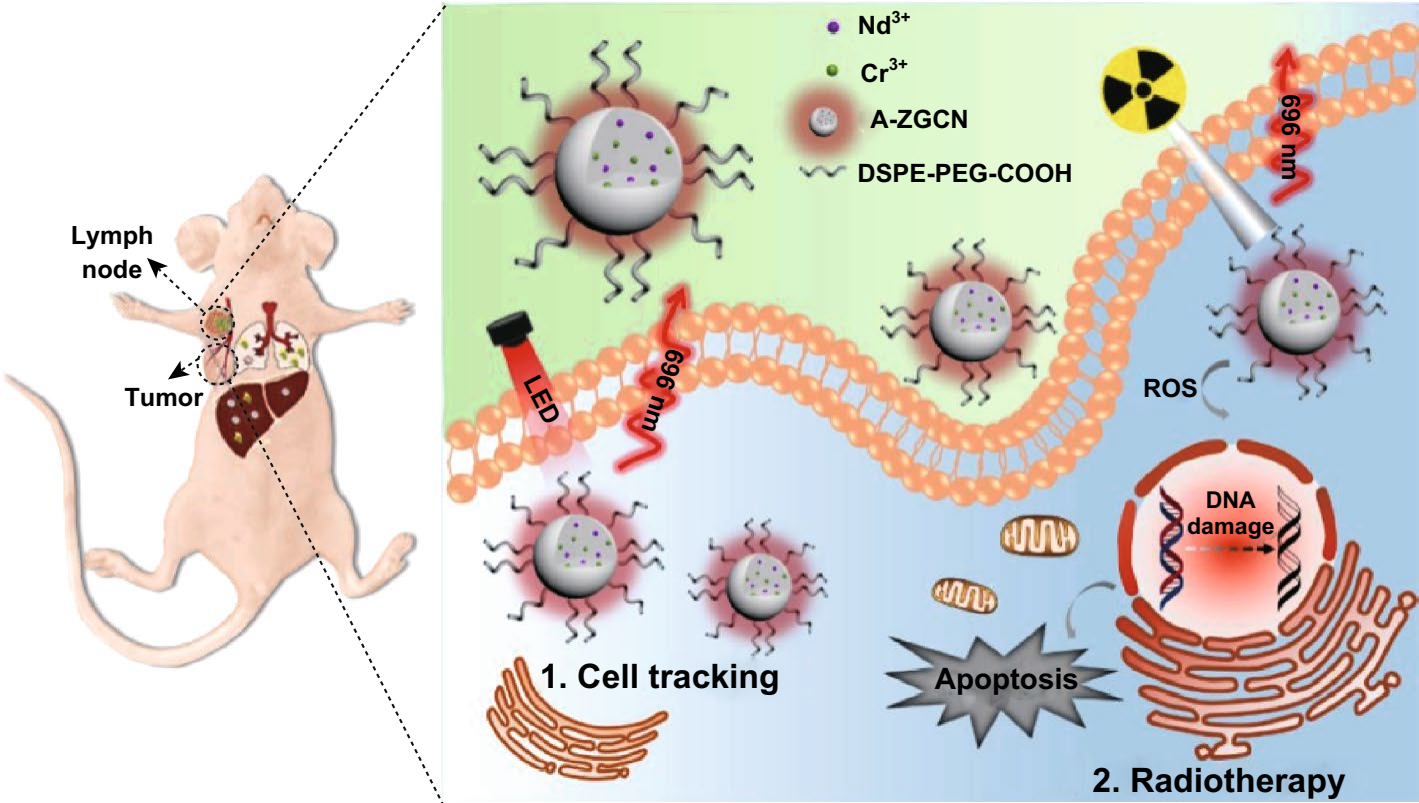
In vivo tracking of breast cancer cells and inhibiting tumor metastasis by radiotherapy with engineered A-ZGCN nanoparticles. Reproduced with permission from Ref. [96]. Copyright 2019 American Chemical Society

We established the first fabrication of TAT penetrating peptide-functionalized PLNPs for the long-term tracking of adipose-derived stem cells (ASC). We used the skin-regeneration and tumor-homing models to study the in vivo tracking and imaging efficiency. PLNPs showed very promising results for ASC tracking without affecting the natural behavior of ASC, which provides us insights to study the fate and migration of ASC [35]. Early detection of cancer metastasis is quite essential and challenging for the cancer diagnosis and treatment. The outstanding advantages of PLNPs in cell imaging show great potential for tracking cancer cell metastasis. Liu et al. reported the PLNPs-based real-time tracking of the orthotopic breast cancer cell metastasis, and its guidance for surgical resection (Fig. 6) [96]. Zhao et al. prepared PLNPs containing a hydrogel (PL-gel) for targeted, sustained and autofluorescence-free tumor metastasis imaging (MBA-MD-231 breast cancer cells). This PL-gel could be rationally designed to target variety of other cancer cells and provide a powerful and versatile method for studying tumor metastasis [97].

PLNPs-based labeling strategies also have universal applications in monitoring other cancer cells. For example, Maldiney et al. reported the first use of biotinylated PLNPs to target the avidin-expressing glioma cells, which provided preliminary results for guiding the use of avidin–biotin technology to target the glioma tumor microenvironment [69]. Zhao et al. conjugated two targeting ligands (hyaluronic acid and folic acid) on the PLNPs, which target specifically toward the cluster determinant 44 receptor and the folic acid receptor on the tumor cells. This dual-targeting strategy provides synergistic effects to improve the specificity and affinity toward the cancer cells [98]. These cellular target imaging and tracking approaches not only inspire studies on the fate of cells in important life activities but also broaden the applications in cell-based research and therapy.

### In Vivo Multimodal Imaging

To date, various imaging techniques have been developed for monitoring various biological processes and visualizing pathogenesis and progression of many diseases, such as the X-ray computed tomography imaging (CT), the magnetic resonance imaging (MRI) and the optical imaging. The PLNPs-based PL imaging (PLI) shows high SNR, easy operation and deep penetrating depth capabilities. Thus, the integration of the PL imaging with other imaging techniques can bridge the gaps in resolution, sensitivity and depth [43]. Several groups have introduced the engineering of multifunctional platforms based on PLNPs for the multimodal imaging (Table 2). The combination of PLI and MRI is most frequently studied [99–103]. These promising nanoprobes as novel diagnosis techniques will open wide applications for biologists and pharmacologists in biomedical research [104–106].

**Table 2:** Typical multimodal in vivo imaging studies with PLNPs

Imaging modalities	Composition of PLNPs	Surface modification	Biological model	Refs.
PL/MRI	Zn_2.94_Ga_1.96_Ge_2_O_10_:Cr^3+^, Pr^3+^	Gd-DTPA	Biodistribution	[99]
PL/MRI	Gd-doped ZGaO:Cr^3+^	PEG	Biodistribution	[43]
PL/MRI	Gd_2_O_3_@mSiO_2_@CaTiO_3_:Pr^3+^	PEG	Biodistribution	[101]
PL/MRI	ZGaO:Cr^3+^@ mSiO_2_-USPIOs	Diglycolic anhydride	Biodistribution	[102]
PL/MRI	Gd_2_O_3_-ZnGa_2_O_4_:Cr^3+^	Hyaluronic acid (HA)	Tumor imaging	[103]
PL/CT	Zn_2.94_Ga_1.96_Ge_2_O_10_:Cr^3+^, Pr^3+^@TaO_*x*_@SiO_2_	Cyclic-Asn-Gly-Arg peptides	Tumor imaging	[104]
PL/MRI/CT	GdAlO_3_:Mn^4+^, Ge^4+^@SiO_2_@Au	–	Tumor imaging	[106]

**Fig. 7:**
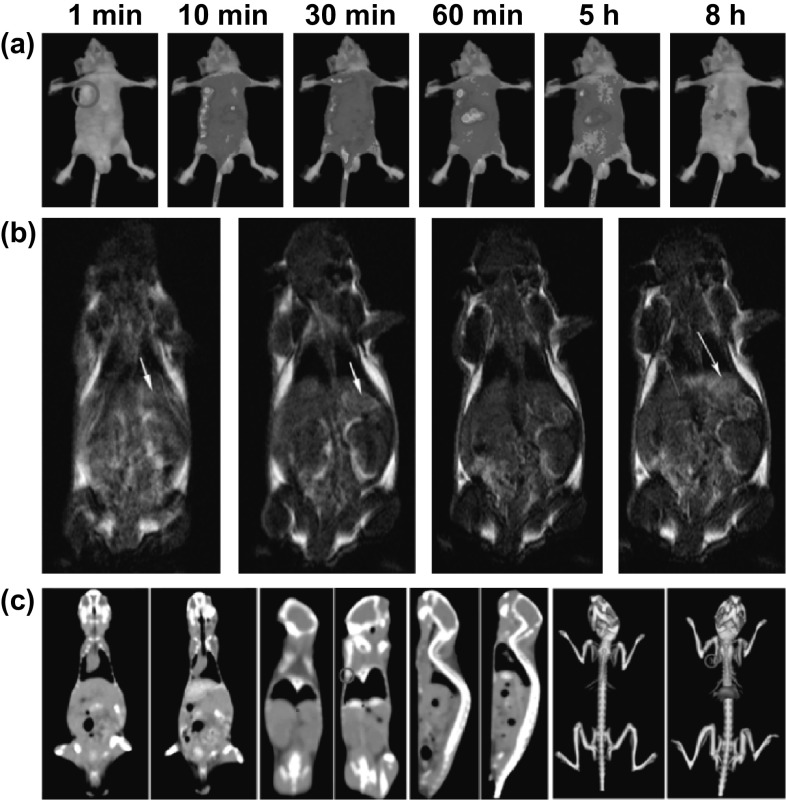
In vivo trimodal bioimaging. **a** PL images at different time points. **b** T_1_-weighted MRI coronal images. **c** CT coronal view images. Reproduced with permission from Ref. [106]. Copyright 2016 American Chemical Society

The in vivo multimodal imaging studies are mostly performed on mice. Maldiney and co-workers introduced the first synthesis and functionalization of a multimodal PLI/MRI nanoprobe based on the gadolinium-doped PLNPs. This novel imaging probe combines the high sensitivity from the PLI with the high spatial resolution of the MRI. The CT serves as a conventional medical imaging technique for a period of time, due to its promising features in high spatial resolution and ease of illustrating 3D biological structures. Lu et al. reported the fabrication of TaO_*x*_ and PLNPs for a PL/CT bimodal imaging. This multifunctional nanoparticle could act as a new potential platform for the tumor imaging with high SNR, low toxicity and good spatial resolution [104]. Liu and co-workers reported GdAlO_3_:Mn^4+^, Ge^4+^@Au (GAMG@Au) core–shell nanoprobes for in vivo tri-modality (PLI/MRI/CT) bioimaging (Fig. 7). The NIR persistent luminescence and the doped Gd element were used for the optical imaging and the magnetic resonance imaging, respectively. The gold nanoshell coated on the PLNPs could not only serve as the CT imaging agent but also enhance the PL efficiency via the plasmon resonance [106].

PLNPs-based optical bioimaging agents have been used in some subcutaneous-tumor bearing mouse models for tumor imaging or biodistribution study. In addition, PLNPs-based nanocomposites could be applied in other important biological models [107]. For example, in the lymphatic imaging, PLNPs could be potentially applied to monitor the location of lymph nodes and study the lymph node functions [108]. We believe that PLNPs are ideal for the long-term monitoring of the considerable biological processes in real time, including the imaging on bacteria, *C*. *elegans* or zebrafish models in the future.

### X-ray Irradiated PLNPs for In Vivo Imaging

**Fig. 8:**
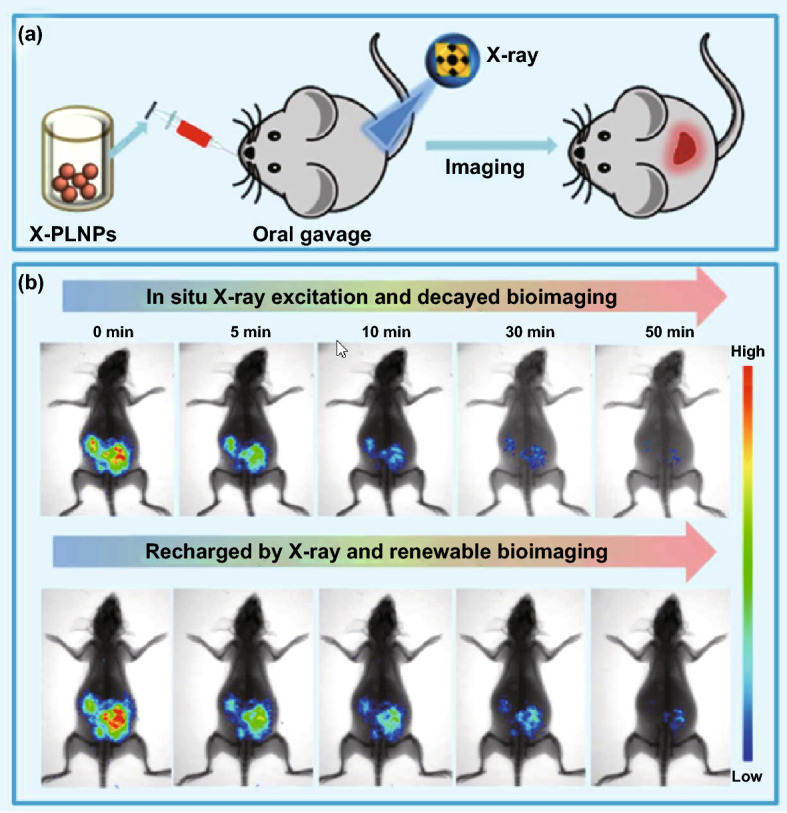
**a** In vivo bioimaging after the oral administration of PEG-modified PLNPs under X-ray irradiation. **b** Rechargeable in vivo whole-body imaging of mouse with oral administrated PLNPs at different time after stoppage of X-ray excitation. Reproduced with permission from Ref. [114]. Copyright 2017 The Royal Society of Chemistry

Currently, the NIR-emitting PLNPs are mainly excited by UV, visible light or red LED light. The relatively short penetration depth of these lights hindered the application for deep tissue imaging. In fact, PLNPs can also be excited by X-ray [109–112]. The development of soft X-ray excitation source with low power in recent years broadens the applications of PLNPs, as it can restore in vivo imaging signals even at 20 mm depth. The NIR-emitting ZnGa_2_O_4_:Cr PLNPs can be repeatedly activated with low-power X-ray. After the intravenous injection or the oral administration, the in vivo whole-body bioimaging was successfully achieved under X-ray irradiation (Fig. 8). Compared to the traditional UV and visible excitation sources, X-ray shows competitive advantages of deeper penetration depth and weaker scattering in tissues which activates PLNPs to emit NIR persistent luminescence for in vivo deeper tissue bioimaging [113–115].

### In Vivo PL Imaging in the Second or Third Biological Window

**Fig. 9:**
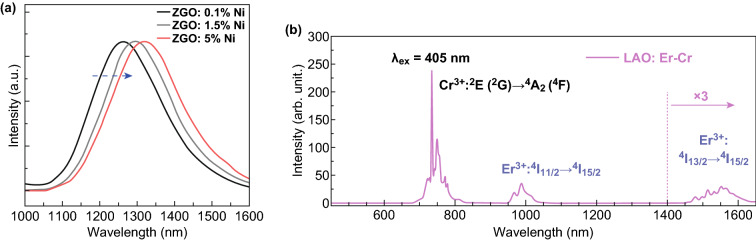
In vivo PL imaging in the second or third biological window. **a** PL spectra in the second imaging window. Reproduced with permission from Ref. [40]. Copyright 2017 Springer Nature. **b** PL spectra in the first and third imaging windows. Reproduced with permission from Ref. [117]. Copyright 2017 The Royal Society of Chemistry

It is well accepted that NIR emission can penetrate biological tissues, such as skin, blood and organs, more efficiently than visible light. However, the light in the second (1000–1400 nm) and the third (1500–1800 nm) NIR biological windows shows the lower absorption, the lower scattering coefficient and the deeper tissue penetration than the light in the first NIR window (650–950 nm). Nie et al. reported new Ni-doped PLNPs with a tunable emission band peaking from 1270 to 1430 nm in the second NIR window (Fig. 9a). These long NIR emitting PLNPs with characteristic operational waveband and excellent tunability offer the possibility for visualizing structural and functional process in cells, tissues and other complex systems [40]. Cr^3+^/Er^3+^-co-doped LaAlO_3_ perovskite phosphors exhibit two long PL bands at 734 (first window) and 1553 (third window) nm (Fig. 9b). These NIR-emitting PLNPs lead to an improved contrast quality and deeper tissue penetration depth than the Cr^3+^-doped PLNPs with the emission in the first NIR window [116–119].

## PLNPs-Based Imaging-Guided Therapy

In recent years, PLNPs have been positively involved in theranostic studies, as the persistent luminescence can be used to determine the accurate position and time that therapy required, alleged “imaging-guided therapy.” Among various therapeutic technologies, chemotherapy, photodynamic therapy (PDT) and photothermal therapy (PTT) are mostly studied due to their obvious treatment effects. With the remarkable penetration depth and biocompatibility, PLNPs can be integrated to provide various PL imaging-guided therapies [120].

### PLNPs-Based Drug Delivery and Chemotherapy

Chemotherapy is one of the most used cancer therapies, although its disadvantages of the drug resistance and the side effects or toxicity on healthy tissues limit the clinical effectiveness. Many nanoparticle-based drug delivery systems have been developed for simultaneous imaging and chemotherapy, which realizes the real-time monitoring of the treatment, the photo-controllable targeting and less side effects. Usually, mesoporous or hollow silica nanoparticles are integrated on the PLNPs as trackable drug carriers due to their large pore structure and specific surface areas [63, 121]. These platforms with high drug storage capacity and excellent NIR persistent luminescence show promising potential for the delivery of any therapeutic agents as the trackable drug carriers.

**Fig. 10:**
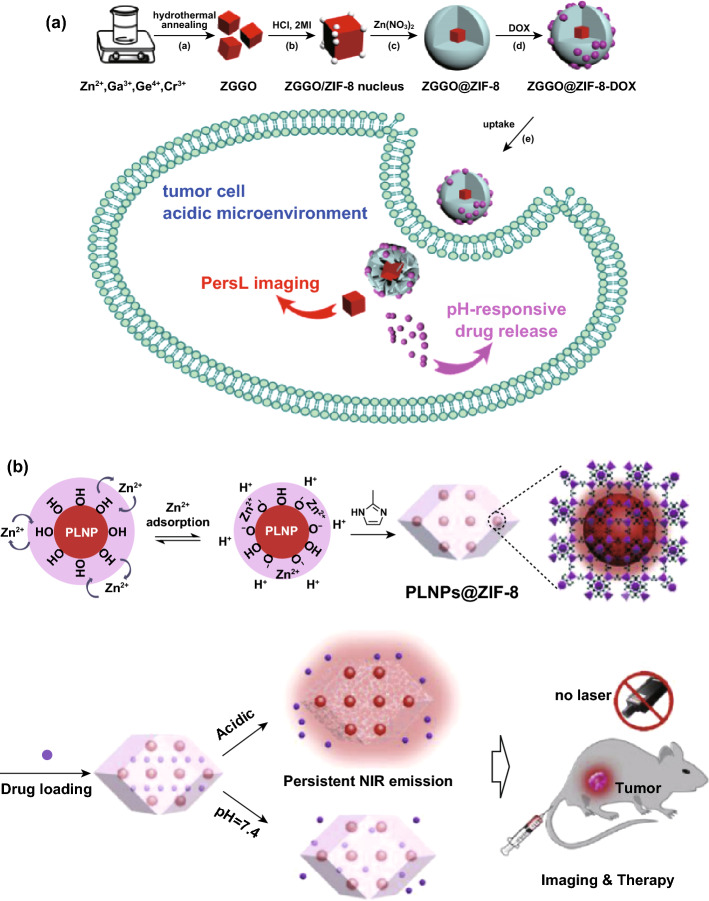
**a** ZGGO@ZIF-8-DOX nanocomplex offered long-term NIR PL signals for the autofluorescence-free bioimaging and pH-responsive drug release for cancer chemotherapy. DOX release was accelerated in the acidic microenvironment of the tumor cells. Reproduced with permission from Ref. [125]. Copyright 2018 American Chemical Society. **b** PLNPs@ZIF-8 for acid-activated tumor imaging and drug release. Reproduced with permission from Ref. [126]. Copyright 2019 Elsevier Ltd

Chen and co-workers established liposome-encapsulated PLNPs as novel PL imaging-guided drug carriers for chemotherapy. Since liposomes have been extensively used as drug carriers for biomedical applications with prominent merits of biocompatibility and biodegradability. The encapsulation of PLNPs in liposomes renders the high drug loading efficiency, long-term NIR emission and remarkable therapeutic capabilities [122]. Mesoporous silica (MS) was another frequently used drug carrier. Zhao et al. showed MS-coated PLNPs with pH-driven targeting and cathepsin B/glutathione dual-responsive drug release capabilities for PL imaging and chemotherapy of tumor [123]. Feng et al. developed a raspberry-like mesoporous Zn_1.07_Ga_2.34_Si_0.98_O_6.56_:Cr_0.01_ (Si-ZGO) nanocarriers for enhanced PL imaging and chemotherapy [124]. Metal–organic frameworks (MOFs) are advanced porous materials constructed by self-assembly of metal ions and multifunctional organic ligands, which have been widely used in separation, catalysis, molecular storage, biosensing/imaging and cancer therapy. The biocompatible porous framework ZIF-8 showed a significant drug loading capacity, which has been used as acidic triggered drug release platform for the efficient cancer chemotherapy. Zhao et al. and Lv et al. constructed a PLNPs@ZIF-8 core–shell multifunctional nanoplatform using different assemble steps and then loaded with doxorubicin (DOX). The tumor site-specific drug release and the persistent luminescence imaging were successfully achieved (Fig. 10) [125, 126]. Apart from these strategies, more efforts need to be made to improve the targeting and therapeutic efficiency [127].

### PLNPs-Based Photodynamic Therapy

PDT is a treatment that uses photosensitizers along with light excitation to generate cytotoxic singlet oxygens (^1^O_2_) to kill cancer cells. Due to its low toxicity and minimal invasiveness to normal cells, it has become an alternative to the conventional therapeutic modality for variety of cancers. However, most photosensitizers for PDT need continuous activation by UV or visible light for a long time, leading to the limitation of the penetration depth in tissues and the irradiation induced overheating and cell damages. PLNPs with long-lasting NIR emission can serve as a persistent light source for PDT activation without any need for continuous photonic excitation [128–130]. This promising feature can minimize the deleterious side effects of PDT and provide convenient clinical cancer treatment without continuous external irradiation [131–133].

Abdurahman and co-workers reported the photosensitizer (Si–Pc) covalently conjugated PLNPs with 808-nm NIR light renewable PL for PDT [131]. Except for covalent binding, Wang et al. [132] prepared a mesoporous silica shell for the photosensitizer (sulfonated aluminum phthalocyanine, a traditional photosensitizer) loading. Fan and co-workers constructed injectable PL implants as a built-in excitation source for an efficient repeatable PDT. This study represents a fresh concept of PLNPs-based PDT, leading to the efficient tumor growth suppression [134].

### PLNPs-Based Photothermal Therapy

**Fig. 11:**
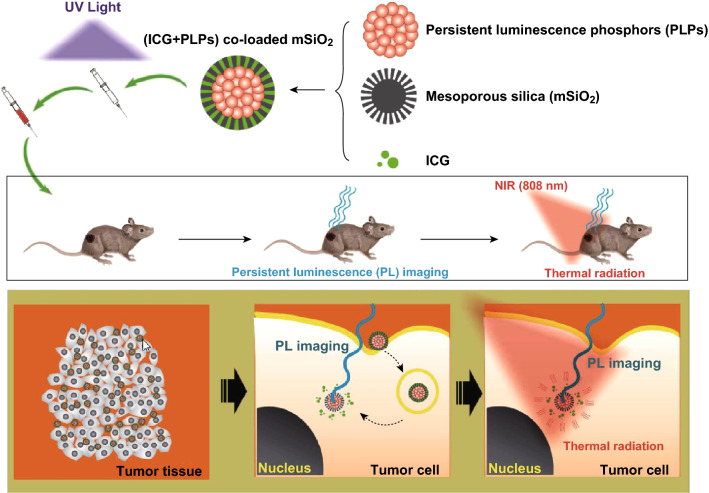
Schematic representation shows the applications of ICG functionalized PLNPs for PL imaging-guided PTT in vivo. Reproduced with permission from Ref. [135]. Copyright 2016 American Chemical Society

In a typical PTT treatment, NIR light is converted into heat by proper agents (gold nanoparticles, CuS nanoparticles, graphene oxides, organic dyes, etc.), resulting in hyperthermia to kill cancer cells. Among the PTT agents, indocyanine green (ICG) is water-soluble and approved by FDA. To further improve the stability of ICG, several works about PLNPs functionalized with mesoporous silica shell have been developed to load ICG for the PL imaging-guided PTT [135–137]. Zheng and co-workers developed the PLNPs and ICG co-loaded mesoporous silica nanoparticles for PL imaging and PTT (Fig. 11). This new PTT agent could be used for significantly killing the cancer cells [135]. Chen et al. designed an activatable multifunctional PLNPs/CuS-based nanoplatform for PL imaging-guided PTT in vivo. CuS nanoparticles served as both PTT agent and the quencher with a high photothermal conversion efficiency and a strong NIR absorption. The prepared nanoprobes showed highly sensitive tumor-targeted PL imaging and effective PTT, leading to great potential for clinical theranostic applications [105].

### Other Imaging-Guided Therapy

In some other cases, PLNPs were combined with other therapeutic agents for multiple applications. We first conjugated therapeutic plasmid on the polyetherimide (PEI)-modified PLNPs. The fabricated dual functional PLNPs can track the engineered mesenchymal stem cells (MSCs) homing and the gene therapy of glioblastoma [138]. Qin et al. [139] designed a gold nanorods/siRNA-assembled PLNPs nanofibers for LED-amplified gene silencing in cancer cells. The controllable integration of PLNPs with new functional groups also shows great potential for other clinical theranostic applications. For example, design of a vaccine/PLNPs-based nanocomposite for effective PL imaging-guided immune therapy was never reported so far [140, 141].

## Biodistribution and Biosafety Evaluations of PLNPs

A growing insight toward in vivo applications has led to major improvements in the evaluation of the biodistribution and biosafety of PLNPs. The influences of the crystal size, the surface modification, the layer thickness and the charge on the biodistribution of PLNPs after intravenous injection have been evaluated in living animals [121, 142, 143]. In general, the tissue distribution is found to be highly dependent on the surface coverage, as well as the core diameter. The PEG grafting is the most widely used surface modification method. It is found that increasing PEG chain length or density on the surface of PLNPs can significantly slow down the uptake process by RES. The precise control of PEG density can prevent protein adsorption on the surface of PLNPs and significantly reduce macrophage uptake in vitro [144]. Such promising results offer broad prospects for long-term applications of PLNPs in vivo.

Toxicity assessment of PLNPs is quite necessary for bioapplications [145, 146]. The in vitro or in vivo evaluation methods include the cytotoxicity study, the cell proliferation and differentiation, the physical and behavioral signs, the histological or hematological analysis, the hemolysis and blood biochemistry analysis and so on. The most convenient approach for evaluation of the cytotoxicity of PLNP is the cell viability test after PLNPs incubation. The impact of PLNPs on the cellular proliferative and differentiative ability is another important indicator of the cytotoxicity. We investigated the influence of PLNPs on the differentiation of adipose-derived stem cells (ASC) and human mesenchymal stem cells (MSCs) by culturing with adipogenic, chondrogenic and osteogenic supplemented medium, respectively. The microscopic images showed no significant stain difference between PLNPs labeled and unlabeled stem cells in each kind of differentiation. Consequently, these data suggest that PLNPs did not affect ASC and MSC differentiation [35, 138].

Physical and behavioral signs, such as the body weight, the excrement analysis, and behavior traits are employed to assess in vivo toxicity. The results generally indicate no significant changes as compared to the control groups [131]. In vivo toxicity of PLNPs is also evaluated via histological studies of main organs, including heart, liver, spleen, lung, kidney and other organs harvested from PLNPs pre-injected mice. The microscopic images of slices stained with hematoxylin and eosin (H&E) showed that PLNPs hardly caused any lesions, tissue damage or inflammation to organs [3, 98]. Ramírez-García and co-workers focused on several parameters, such as reactive oxygen species (ROS) indexes upon exposure to PLNPs, alterations in morphology at tissues and cellular levels and impact on blood cell counts [145, 146]. However, the currently obtained results of the biosafety evaluation of PLNPs are limited by relatively rudimentary and superficial methods. The possible immunotoxicity introduced by PLNPs needs further research in vivo. The relationship between the characteristics of PLNPs (composition, shape, size, surface modification, etc.) and toxicity needs to be systematically and profoundly established, especially for the long-term tracking studies.

## New Organic and Polymeric PLNPs with Long Afterglow for In Vivo Optical Imaging

Nowadays, the number and type of PLNPs are still relatively limited. The main interest worldwide has focused on the rare earth heavy metal ions-doped inorganic materials. The high-cost, relatively complicated preparation and surface modification methods present the barriers for commercialization. Therefore, the development of new types of inexpensive, long-term emitting, biocompatible, eco-friendly and heavy metal ions-free afterglow materials is highly desirable [147–149]. Yang et al. reported a new type of metal–organic frameworks (MOFs) with long-lasting afterglow which showed highly tunable afterglow phosphorescence colors upon pyridine solution treatment. This finding supplies a group of MOFs-based persistent luminescence nanophosphors with high performance [150–154]. Up to now, the afterglow MOFs have not been used in biomedical fields in vivo due to the relatively short emission wavelength and the undesirable diameter.

**Fig. 12:**
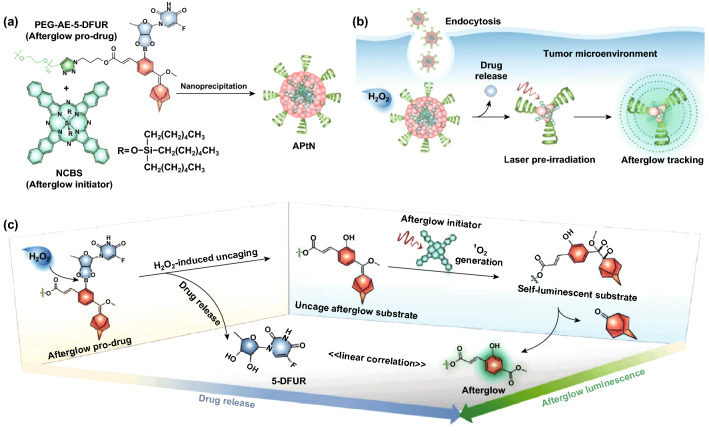
**a** Design and synthesis of APtN. The chemical structures of the afterglow initiator (NCBS) and the afterglow pro-drug (PEG-AE-5-DFUR). **b**, **c** Tumor microenvironment responded drug release and laser irradiated ^1^O_2_ generation and afterglow tracking. Reproduced with permission from Ref. [163]. Copyright 2019 WILEY–VCH Verlag GmbH & Co. KGaA, Weinheim

In recent years, afterglow organic ingredients and polymers have captured special attention of chemist and biomedical scientists [155–157]. Semiconducting polymer nanoparticles (SPNs) assembled from completely benign organic components have emerged as versatile optical agents for molecular imaging [158–160]. Miao et al. presented the SPNs that can store the photo energy via chemical defects and emit NIR long persistent luminescence at 780 nm. The afterglow intensity of the SPNs show more than 100-fold brighter than that of inorganic PLNPs. SPNs were used for the lymph node and tumor imaging with a significant high SNR. Moreover, the developed SPNs-based probe can detect the early drug-induced hepatotoxicity in living mice [42]. Recently, He et al. have developed an organic afterglow protheranostic nanoassembly (APtN) with the afterglow imaging and the drug release in response to tumor microenvironment (excessive H_2_O_2_). Such molecular architecture combines passively tumor targeting, specific drug releasing and spontaneous afterglow generation, which provides design guidelines for activatable cancer theranostics (Fig. 12) [161–163].

**Fig. 13:**
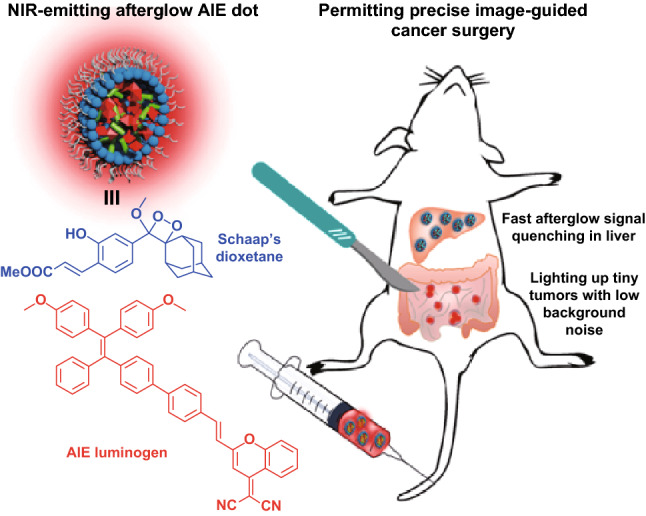
NIR-emitting afterglow AIE dots for precise image-guided cancer surgery. Reproduced with permission from Ref. [164]. Copyright 2018 American Chemical Society

The organic and polymeric PLNPs show high biocompatibility, good biodegradability and flexible synthesis and surface modification advantages for bioimaging and imaging-guided therapy. So far, there is rare report on the precise in-depth imaging-guided cancer surgery. Ni et al. synthesized a NIR PLNPs with aggregation-induced emission (AIE) characteristics. This AIE PLNPs showed innate property of fast PL signal quenching in normal tissues and gave ultrahigh tumor-to-liver signal ratio. These fascinating features make AIE PLNPs an excellent imaging-guided probe for peritoneal carcinomatosis resection (Fig. 13) [164]. These new types of persistent luminescence nanophosphors with NIR emission, biocompatible and biodegradable nature have great promise as advanced molecular imaging tools, while the mechanism, synthesis methods, toxicity, imaging sensitivity and wide applications need further exploration and achievements.

## Conclusion and Outlook

In this review, we provided a full-scale review of PLNPs from fundamental principles to all possible applications. The PLNPs possess a special advantage in eliminating autofluorescence interferences without constant in situ excitation, which is ideal for long-term bioapplication. We discussed the main and recent developments in the diagnostic and therapeutic applications of PLNPs, covering biosensing, multimodal imaging, drug delivery and imaging-guided therapy. In order to promote the biomedical applications of PLNPs, general and economic protocols for the synthesis and surface modification have been developed, which are optimized for bioapplications with high biocompatibility, stability and low toxicity. PLNPs with different composition have been widely designed to enhance the PL intensity and the decay time to achieve better in vivo imaging performance in the past decade, while, as shown above, not all of the reported routes can simultaneously control the shape, size and homogeneity of nanoparticles as well as the long persistent luminescence duration at the same time. The involved methods just partly improve the properties of PLNPs. Although rapid progress has been made in synthesis process, there are still many fields that need additional work, including controllable synthesis of PLNPs with new wavelength emission and excitation bands, improving phosphorescence lifetime and afterglow intensity, exploring new activators, matrices and novel multifunctional application fields.

Several pioneering works on investigating the biosensing applications of PLNPs have been reported. The PL-based biosensing can reveal analysis of biomolecules with superior SNR and high sensitivity in complicated biological samples. It is highly desirable to develop PLNPs-based bioprobes for monitoring other important biomolecules in vivo, such as the levels of toxins and signal molecules in the living body. PLNPs with multiple combinations and modifications offer more possibilities for incorporation with other imaging modalities (MRI, CT, PET, etc.). PL imaging-guided therapy can afford guiding cancer therapy with superior SNR. PLNPs can emit long persistent luminescence without continuous in situ external excitation and can act as the internal light source for imaging-guided therapy, avoiding the overheating and tissue damage caused by conventional photo-assisted therapies with constant light excitation (UV light, 808 nm light, etc.).

The various approaches for biosafety assessment of PLNPs have also been briefly studied. PLNPs show great promise in bioapplications without obvious toxicity. For clinical applications, more efforts need to be devoted beyond nanoplatform construction, such as efficacy, price, clinical safety and degradation. Very recently, Lécuyer et al. have studied the degradation of PLNPs in biological media mimicking solutions. They provided valuable information for the possible elimination of PLNPs after in vivo preclinical applications [165]. The PL imaging-guided tracking of the disease processes should also be studied in the future. We hope that this review could comprehensively summarize the properties and bioapplications of PLNPs and will shed new light on future directions to develop novel PLNPs and discover novel benefits for multiple applications.
